# Patient experience and psychologists' attitudes toward AI in psychological treatment: A topic-modeling study

**DOI:** 10.1016/j.invent.2026.100999

**Published:** 2026-09-18

**Authors:** Jón Ingi Hlynsson, Kristofer Vernmark, Catharina Lutovsky, Gerhard Andersson, Per Carlbring

**Affiliations:** a Department of Psychology, Stockholm University, Stockholm, Sweden; b Department of Behavioural Sciences and Learning (IBL), Linköping University, Linköping, Sweden; c University College, Korea University, Seoul, South Korea; d Department of Clinical Neuroscience, Karolinska Institute, Stockholm, Sweden; e Lusófona University, Digital Human-Environment Interaction Labs, Lisboa, Portugal

**Keywords:** Artificial intelligence, Psychotherapy, Social anxiety disorder, Topic modeling, Psychologist attitudes, Patient experience, Virtual patients

## Abstract

Generative artificial intelligence (AI) is entering psychological treatment, yet research remains dominated by symptom outcome trials, leaving two questions under-researched: how patients experience AI therapy tools, and how psychologists regard the AI applications they may be asked to adopt. We applied topic modeling (Latent Dirichlet Allocation) to two Swedish-language corpora to contrast lived use with anticipated use. The first corpus comprised interview data from 11 patients who had engaged with a conversational AI assistant in the treatment of social anxiety disorder. The second comprised 1551 open-ended survey responses from 808 psychologists (licensed psychologists, psychologists in clinical internship, and psychology master's students) with analyzable text for at least one of two scenarios: 1) a psychologist's “*Digital Twin*” (between-session avatar), and 2) AI “*Virtual Patients*” for clinical training. Text was lemmatized, and a four-topic solution was selected for each corpus based on interpretability and lexical distinctness. Patients described using the AI to analyze social situations and to work through difficult feelings. They valued its privacy and constant availability, said it let them avoid the stigma of seeking help, and compared it throughout with a human therapist. Psychologists expressed enthusiasm about the use of Virtual Patients as a training tool, whereas the Digital Twin scenario raised questions about authorship and responsibility. Concerns about relational loss and the balance between utility and risk were expressed across both scenarios, and both patients and psychologists noted that AI could not replace a human relationship. Given the small patient sample, these findings are exploratory and hypothesis-generating.

## Highlights

• Topic modeling of patient interviews after AI-delivered therapy for social anxiety
• 808 psychologists reacted to Digital Twin and Virtual Patient future scenarios
• Patients valued private, on-demand access that sidestepped help-seeking stigma
• Psychologists accepted AI as a stand-in for patients, not for themselves
• Both groups placed the ceiling of AI at the human relationship it cannot provide

## 1 Introduction

The integration of artificial intelligence (AI) into mental health care has accelerated with the arrival of large language models (LLMs). AI is the broader category, while generative AI specifically creates new content from user prompts. LLMs are the foundational models trained on extensive text datasets, and chatbots are the conversational interfaces that utilize an LLM to interact with users (Thirunavukarasu et al., 2023). Whereas early therapeutic chatbots delivered scripted, rule-based content (e.g., Woebot; Fitzpatrick et al., 2017), contemporary generative systems produce open-ended, conversational responses that more closely resemble unstructured clinical dialogues. Early trials of such systems report reductions in symptoms of depression and anxiety (e.g., Heinz et al., 2025; Hlynsson et al., 2026; see Zhang et al., 2025 for a meta-analysis), and taxonomies of these applications have begun to emerge (Schuster et al., 2026). This shift raises questions that symptom outcome trials alone cannot answer. How patients and psychologists experience and evaluate generative AI therefore becomes important as AI moves from a delivery mechanism for fixed content toward an interactive agent.

Generative AI developments extend an established tradition of technology-assisted psychological interventions. Technology use in psychotherapeutic interventions has grown rapidly over the past four decades, with a bibliometric analysis indicating that research output approximately tripled across successive decades (Zale et al., 2021). Within this tradition, guided and unguided internet-based cognitive behavioral therapy (ICBT) has accumulated decades of efficacy evidence (Hedman-Lagerlöf et al., 2023; Vernmark et al., 2024; Winter et al., 2023). AI-supported variants are now being embedded in the same treatment platforms through a *compartmentalized architecture*, in which de-identified data are processed externally and the resulting outputs are checked by a clinician before they are sent to participants (Vlaescu et al., 2026). Advances in AI technology may automate parts of therapeutic guidance through always-available psychoeducational coaching and clinically informed feedback (Carlbring et al., 2023; Wiederhold, 2025), enabling on-demand support beyond office hours and reducing waitlists. Positioned this way, generative AI is a further step from bibliotherapy through therapist-guided ICBT to automated support, offering on-demand contact between sessions while retaining the scalability rationale behind internet interventions (Löchner et al., 2025).

Social anxiety disorder (SAD) can be a suitable diagnosis for the question of how to implement generative AI into clinical practice. SAD is defined by a marked fear of negative social evaluation (American Psychiatric Association, 2022; Clark and Wells, 1995), and the social-evaluative concerns that maintain the disorder also affect help-seeking itself. Most individuals with SAD do not seek treatment (Patel et al., 2022), stigma predicts weaker help-seeking intentions (McDonagh et al., 2022), and the median delay to first treatment contact is roughly 16 years (Wang et al., 2005). Tools that can be accessed privately, anonymously, and without the immediate presence of another person may therefore be suitable for this population, and there is emerging evidence that help-seeking self-stigma pushes some individuals toward AI-mediated alternatives (Békés and Aafjes-van Doorn, 2026; Hoffman et al., 2024). SAD thus offers a setting in which the core features of AI-driven therapy (i.e., privacy, availability, and the absence of a human conversational partner) map directly onto the clinical features of the disorder. However, this same fit carries a risk. Contact that never exposes the patient to another person can itself become a safety behavior, the kind of subtle avoidance that cognitive models identify as maintaining social fear (Clark and Wells, 1995).

Despite rapid adoption, the evidence base for AI-mediated therapy remains dominated by symptom-oriented outcome trials (e.g., Zhang et al., 2025) which cannot capture everything at stake. Recent work evaluating LLMs against clinical standards has shown that LLMs can express stigma toward people with mental health conditions and respond inappropriately in safety-critical situations (Moore et al., 2025), which symptom measures would not detect. What remains under-researched is how the people on either side of these tools understand them. Patients' subjective experience shapes engagement and acceptability, yet it is rarely examined directly (though see Siddals et al., 2024). Psychologists, as the gatekeepers and prospective providers of these tools, bring professional and ethical judgments that will determine adoption (Mackey, 2026; Vernmark et al., 2024).

Across the available literature, AI is examined from a perspective that participants imagine instead of examining AI that they have actually used (e.g., Hipgrave et al., 2025), yet attitudes toward a hypothetical system may diverge from the experience of an actual one (Lee et al., 2025). Furthermore, patient and clinician perspectives are typically reported in separate studies, using different instruments and analytic traditions, so they are rarely brought into contact even when they concern the same technology (e.g., Heinz et al., 2025; Mirzaei et al., 2024). A partial exception is Southwick et al. (2024), who applied LDA to open-ended responses from patients and therapists in the same trial; the present study extends that design to two groups whose exposure to the technology differs.

This study comprises two complementary analyses. In the first, we examine open-ended interview data from patients who received an AI-supported psychological treatment (psychodynamic and cognitive behavioral arms) for SAD within a randomized controlled trial (Hlynsson et al., 2026), asking how they experienced the treatment. In the second, we analyze open-ended survey responses from psychologists responding to two contrasting future scenarios: an AI “*Digital Twin*” of the psychologist for between-session support, and AI-simulated “*Virtual Patients*” for clinical training (Vernmark et al., 2026). We treat the difference between actual and anticipated use as itself informative. Our analyses therefore address three questions: (a) how patients characterize their experience of an AI therapy tool, (b) how psychologists reason about prospective AI applications, and (c) where the perspectives of those who have used such a tool converge with or diverge from those of psychologists anticipating its future use.

## 2 Method

### 2.1 Study design

This study combines an interview component with a separate cross-sectional online survey component. We analyzed the two Swedish-language text corpora separately using LDA and compared their interpretation. The patient interviews concern an application participants had used; the survey concerns hypothetical applications. We use COREQ (Tong et al., 2007) and CROSS (Sharma et al., 2021) to organize reporting of the interview and survey components, respectively.

### 2.2 Patient interviews: participants and data collection

The patient dataset comprised transcripts from 11 patients who had participated in a Swedish-language randomized controlled trial of AI-delivered psychological treatment (psychodynamic and cognitive behavioral arms) for social anxiety disorder (Hlynsson et al., 2026). Participation in the interviews was voluntary, and respondents provided informed consent through the online platform (Vlaescu et al., 2026). Transcripts were collected in Swedish. Interviewer turns were removed before preprocessing, so the corpus comprises patient turns only, yielding a total of *n* = 443 patient segments contributed by the 11 participants (median = 38 segments per participant, range 31–61), of which 390 were analyzable after removing segments left empty when infrequent terms were pruned from the document-term matrix (Table S1). Patient demographic characteristics are summarized in Table 1.

**Table 1:** Demographic data for interviewed participants.

Age	M = 43 years (22–57)
Gender	9 women, 2 men
Occupation	9 employed, 2 students
Type of therapy	6 CBT, 5 PDT
LSAS-SR pre-treatment	M = 66 (45–102)
LSAS-SR post-treatment	M = 52 (26–89)

Note. *M* = mean. Ranges are provided in parentheses. CBT = Cognitive Behavioral Therapy; PDT = Psychodynamic Therapy; LSAS-SR = Liebowitz Social Anxiety Scale–Self-Report.

In the parent trial, human therapist support was replaced by an AI assistant providing reflective questions, reminders, and encouragement within psychodynamic or cognitive behavioral treatment (Hlynsson et al., 2026). The system used Claude 3.7 Sonnet for all agents in a multi-agent architecture with retrieval-augmented generation. Participants could use it on demand, up to 75 messages per day. Conversations were encrypted locally without central logging, and participants could flag responses for review by a licensed psychologist. We describe this application as an LLM-based conversational system, referred to below as the AI assistant. References to AI in the patient findings concern this particular system.

Only participants randomized to AI-CBT or AI-PDT were shown the electronic interview invitation at post-treatment assessment. Twenty expressed interest, 14 replied to follow-up contact, and three preferred text-only participation; 11 completed telephone interviews. The recruitment target was 15, and availability rather than a formal saturation rule determined the final sample.

Catharina Lutovsky, a female psychology student in a five-year clinical psychology program with prior qualitative-interview training and experience, conducted one telephone interview per participant from her quiet home in May and June 2025. Interviews averaged 20 min (range 12 to 28), were recorded with consent, and were transcribed using Whisper in autumn 2025 with manual correction. The supplementary material reports interviewer characteristics, relationships, reflexivity, guide development, field notes and the absence of participant checking. The Swedish interview guide and English translation are also included in the supplementary material.

### 2.3 Psychologist survey: participants and data collection

A separate cross-sectional survey of psychologists was conducted to assess attitudes toward hypothetical future applications of generative AI in clinical practice. The sample described here comprises the 818 respondents who provided scenario text. The full recruitment and exclusion flow is reported below. The sample comprised licensed psychologists, psychologists completing the postgraduate clinical internship (PTP), and psychology master's students. For brevity, we refer to this group as psychologists throughout. Participants completed an online questionnaire in which they gave open-ended responses to two hypothetical vignettes (see scenarios below). This format was chosen to capture the full range of spontaneous reasoning and valenced responses, without constraining participants to predefined response categories.

The two vignettes were designed to represent qualitatively distinct modes of AI integration: one positioning AI as a surrogate for the psychologist (Digital Twin), and one positioning it as a training tool (Virtual Patients). Each was presented as a near-future scenario followed by a prompt for the participant's immediate reactions. The survey vignettes specified generative AI applications without naming an underlying model. The Digital Twin scenario concerned an interactive avatar for clients, whereas the Virtual Patients scenario concerned simulated clients for professional training. These were hypothetical applications rather than evaluations of the system used by the patient sample.

The questionnaire was developed by Kristofer Vernmark with expert feedback from Gerhard Andersson and Per Carlbring. It was administered in Swedish through Iterapi; no separate participant pretest was conducted. This analysis concerns its two scenario prompts, presented in fixed order with optional free-text fields. Other questionnaire components are reported separately (Vernmark et al., 2026). Instrument development, administration and reporting details are provided in the supplementary material. The online questionnaire was open for 41 days in January and February 2026. Convenience recruitment used the Swedish psychology association's email and social-media channels, with expected overlap between audiences (Vernmark et al., 2026). Further information about participation, consent and recruitment contacts is detailed in the supplementary material.

In the parent survey, 1130 people provided consent and 1084 started the questionnaire. Data cleaning and visual inspection included checks for duplicate responses, atypical timestamps and straightlining; seven respondents were excluded, leaving 1077 in the parent sample (Vernmark et al., 2026). These counts describe the parent survey and should be distinguished from the scenario-response sample and document-preprocessing flow reported below. The scenario module was offered as an optional continuation. No separate sample-size calculation, dedicated nonresponse analysis or population weighting was performed for this secondary free-text analysis. A reliable denominator of unique invitees was unavailable, precluding a conventional overall survey response rate. Of the 1077 respondents to the parent survey, 824 continued to the optional scenario module and entered text in at least one scenario field. Six entered only non-substantive characters (e.g., stray punctuation) in both fields and were excluded prior to any document preprocessing, leaving the 818 psychologists reported here. The scenario analysis included 818 respondents before document preprocessing. Two scenario fields were blank and another 83 were emptied of content words during preprocessing (Table S4). Table 2 describes the 818 respondents before document preprocessing; 808 contributed analyzable text. Valid age was missing for 24 respondents and year of licensure for 118.

**Table 2:** Demographic data for the surveyed psychologists.

Age in years, M (SD) [range]	45.8 (12.6) [24–79]
Gender, n (%)	
Woman	531 (64.9)
Man	274 (33.5)
Other	7 (0.9)
Not reported	6 (0.7)
Years since licensure, M (SD) [range]	14.6 (10.4) [0–55]

Note. The table describes the 818 respondents retained before document preprocessing. Age was missing or outside plausible bounds for *n* = 24; year of licensure was missing or outside plausible bounds for *n* = 118 (values set to missing). In the parent survey sample, 901 of 1077 respondents (83.7%) were licensed psychologists. This percentage describes the parent survey, not the scenario-response subsample.

#### 2.3.1 Scenario 1 (digital twin)

Imagine a future scenario in which generative AI makes it possible for you, as a psychologist, to create a “*digital twin*” or avatar version of yourself. Clients could interact with this avatar (e.g., asking questions, discussing, receiving support, or being guided through exercises) between your regular sessions or when you are unavailable. What are your immediate thoughts and reflections on such a possibility for clinical work?

#### 2.3.2 Scenario 2 (virtual patients)

Imagine a future scenario involving “*virtual patients*,” in which generative AI simulates specific clinical situations; for example, a client with a psychiatric diagnosis, a person in crisis, or someone struggling with motivation during ongoing treatment. The simulation allows practice in, for instance, diagnostic interviews, working with the therapeutic alliance, or delivering specific interventions. What are your immediate thoughts and reflections on using such AI simulations for the training and continuing professional development of psychologists?

### 2.4 Ethics

The interview study was covered by approval from the Swedish Ethical Review Authority (Dnr: 2025-01603-02). The Authority determined that the psychologist survey containing the two scenarios fell outside the scope of mandatory ethical review (Dnr: 2025-00064-01). Participants in both studies provided informed consent before taking part, and all analyses were conducted on pseudonymized data. Research data were stored in SUNET Drive with two-factor authentication, with access restricted to authorized research-team members and no public sharing links. Interview audio recordings were deleted to protect participants' privacy. Transcripts were retained with a retention period of 10 years.

### 2.5 Data analysis

Both corpora were analyzed using topic modeling, which identifies recurring patterns of word co-occurrence and represents them as topics. We used Latent Dirichlet Allocation (LDA), a probabilistic topic model distinct from the LLM-based treatment system, to identify the underlying thematic structures. Originally developed by Blei et al. (2003), LDA has since been widely applied to open-ended text data in health and social science research as a method for surfacing latent meaning structures that are difficult to detect through manual coding alone (see e.g., Hagg et al., 2022; Leigh et al., 2025). The output was validated qualitatively by reviewing high-probability terms in their original context.

Swedish text was preprocessed in R (R Core Team, 2024) using textmineR (Jones et al., 2025) and udpipe (Wijffels and Straka, 2026). Lemmatization replaced stemming; lowercasing, removal of punctuation and numbers, and one lemma-aware stop list were applied identically to both corpora. Models used the original Swedish text; quotations were translated only after analysis. A document-term matrix included unigrams, bigrams and trigrams, excluding terms appearing in fewer than two documents. LDA used Gibbs sampling (2000 iterations; alpha = 0.1, beta = 0.5). We evaluated k = 2 to 15 and selected k = 4 for interpretability and lexical distinctness rather than maximum coherence (Chang et al., 2009). The coherence maxima occurred at k = 7 for patients and k = 10 for psychologists (Tables S3 and S5). Interpretation used high-probability terms and the three highest-loading documents per topic subject to a 15-content-token floor. Full preprocessing details, selection rationale and validation procedures are provided in the supplementary material.

We use theme to denote the interpretive label assigned to an LDA topic. Per Carlbring and Jón Ingi Hlynsson reviewed the model output and high-loading excerpts in context and agreed on interpretations through discussion. There was no independent double coding or inter-rater reliability statistic. Earlier manual coding did not inform these interpretations and no separate formal negative-case analysis was performed. All models used a fixed random seed. Topic prevalence was the mean posterior document-topic probability with equal document weighting, not the proportion of participants or documents assigned to a topic. These are descriptive estimates, without inferential confidence intervals or between-group significance tests. Estimates used 390 patient segments from 11 participants and 1551 documents from 808 psychologists (783 Digital Twin and 768 Virtual Patients). Seed stability was stronger for the psychologist solution than for the patient solution; a biterm model provided a short-text benchmark (Yan et al., 2013). Coherence, lexical distinctness, seed stability and document flow are detailed in the supplementary material.

## 3 Results

### 3.1 Patient sample

The Latent Dirichlet Allocation (LDA) analysis of the patient transcripts (*n* = 443 interview segments from 11 participants; 390 analyzable) yielded four interpretable thematic clusters. Given the small sample, this analysis is exploratory and hypothesis-generating. Topic prevalence was stable whether computed at the segment or participant level (differences ≤0.5 percentage points), and all 11 participants contributed to every topic (largest single-participant share of any topic's total posterior mass across the 390 segments ≤19%), indicating the themes are distributed across the sample rather than driven by individual speakers. Table 3 outlines the thematic labels, mean topic weights across 390 segments from 11 participants, and top terms for each topic. An additional example for each theme in the patient sample is provided in the supplementary material.

**Table 3:** Latent Dirichlet Allocation (LDA) Topic Overview: Themes, prevalence, and top terms identified in patient transcripts (k = 4). Prevalence is the mean posterior topic weight across 390 analyzable segments from 11 patients, expressed as a percentage.

Topic	Thematic label	Prevalence	Top terms	Top terms
			(Swedish)	(English Translation)
1	Analytic Engagement & Situational Insight	25.1%	ai, direkt, övning, terapeut, jobb, känd, jobba, prova, nå, situation	ai, directly, exercise, therapist, work, familiar, to work, try, reach, situation
2	Deep Clinical Processing & Self-Understanding	22.3%	situation, social, ångest, förstå, social_ångest, känna, hjälp, fråga, dels, tidig	situation, social, anxiety, understand, social_anxiety, feel, help, ask, partly, early
3	Relational Authenticity, Stigma & the Human-AI Comparison	25.0%	känna, ai, människa, svar, terapeut, förstå, hjälpa, använda, insikt, fråga	feel, ai, human, answer, therapist, understand, help, use, insight, ask
4	On-Demand Access & Engagement Logistics	27.6%	känna, skriva, gärna, samtal, nästan, svår, fråga, lägga, telefon, jobbig	feel, write, gladly, conversation, almost, difficult, ask, put, telephone, tiresome

#### 3.1.1 Theme 1: analytic engagement & situational insight

The first theme centered on the AI's utility as an active, tactical tool for deconstructing social interactions. High-loading terms in this cluster included situation, exercise, try, and directly. Rather than passive listening, the AI provided analytical frameworks that patients found highly engaging, sometimes to the point of hyper-focus. One patient mentioned that:

“*It becomes a completely different type of follow-up with the AI. It is like a direct follow-up: you have something to work on, you encounter it, you reflect on it with your AI, and you adjust it directly. That never really happens with a psychologist, where it might be weeks [between sessions].*”

The persistence of engagement-oriented language across transcripts in this cluster suggests that the interactive, insight-generating quality of the AI was experienced as intrinsically motivating, to the point where some patients described continuing well beyond their initial intention.

#### 3.1.2 Theme 2: deep clinical processing & self-understanding

The second theme captured the affective dimension of patient-AI engagement. Transcripts in this cluster were dominated by language of emotional disclosure and self-directed work, with patients describing the AI as a non-judgmental space in which to deeply explore feelings of anxiety, with high-loading terms centering on anxiety and understanding. One patient pointed to the AI's “*unfathomable patience*,” describing how explanations could be repeated “*over and over again*” until a deeper insight was reached. Several patients also described gaining explanatory models of their own physiological and psychological reactions.

“*Above all, that I understand it [my social anxiety] better... what triggers me and why. We have basically gone back to my school days and childhood, and also compared with other people. I think it has been helpful to get feedback when I've compared my reactions with others, and to have it explained what the difference in perception is that causes someone to get triggered by something that doesn't affect me at all, and vice versa. I would say that the social anxiety has improved.*”

Patients actively discussed using the AI as a tool to try new approaches and challenge some of their thoughts and beliefs related to their social anxiety problems.

#### 3.1.3 Theme 3: relational authenticity, stigma & the human-AI comparison

Despite the benefits, patients maintained a critical awareness of the artificiality of the interaction. Transcripts in this cluster were heavily loaded with terms contrasting the AI with a human therapist. Contextual review revealed friction regarding the AI's limited intuitive understanding and a fear of stigma, leading patients to hide the therapy from others. For instance, one patient mentioned that:

“*I haven't told my friends that I've been working with an AI therapist, and I think they would question it. It's not something I personally feel like discussing. If it helps me, then it works, you know. And I feel... no, I actually haven't told anyone. But I have talked to the AI therapist about it, about this stigma, like, around AI therapy...*”

When contrasting the AI interaction with a human-to-human interaction, another patient noted that the AI felt unnecessarily upbeat and noticeably artificial.

#### 3.1.4 Theme 4: on-demand access & engagement logistics

This cluster centered on the logistical advantages and inherent adaptability of treatment brought about by having the AI assistant always available. Traditional scheduled therapy was replaced by the ability to write to the AI as needed. This also meant that patients could adapt the pace of treatment to their schedules and engagement at any given time. For instance, one patient described it as:

“*Either I felt that I had talked things through, or it came naturally from something we had discussed and Simon [the AI] suggested what I could try; if we agreed that I should do it, that became a natural end to it.*”

Patients also emphasized the value of readily accessing support during off-hours, particularly in the evening, to ask questions and keep trying in difficult moments in real-time, rather than “*storing up*” issues for a weekly appointment.

### 3.2 Psychologist sample

The 818 psychologists had two scenario response fields each, yielding 1636 records before preprocessing. Of these, 85 were excluded (2 blank and 83 emptied of content words during preprocessing), leaving 783 Digital Twin and 768 Virtual Patients documents from 808 psychologists, 743 of whom contributed analyzable documents to both scenarios. These documents were analyzed with Latent Dirichlet Allocation, parallel to the patient analysis and using the same lemma-aware stoplist and model settings. A four-topic solution was retained on the grounds set out in Data Analysis; each topic corresponded to an interpretable response pattern, two of them anchored to a scenario and two cutting across both. After lemma normalization, the two most similar topics shared 5 of their ten highest-probability terms, or 1 term once the corpus-wide terms *ai*, *känna*, *patient*, and *psykolog* were excluded, and no pair of topics exceeded a cosine similarity of 0.55 between topic-term distributions.

Table 4 reports the four topics, their mean topic weights across 1551 documents from 808 psychologists, and top terms. By-scenario prevalence is reported in the text. Two topics were strongly scenario-anchored.

**Table 4:** Latent Dirichlet Allocation (LDA) Topic Overview (Scenarios). Prevalence is the mean posterior topic weight across 1551 analyzable documents from 808 psychologists, expressed as a percentage. Values may not sum to 100% because of rounding.

Topic	Thematic label	Prevalence	Top terms (Swedish)	Top terms (English translation)
1	Training Utility & Skill Rehearsal	31.2%	låta, utbildning, positiv, patient, träna, rollspel, jättebra, psykolog, möjlighet, verklig	sound, education, positive, patient, train, role-play, great, psychologist, opportunity, real
2	Conditional Acceptance & Clinical Authorship	24.6%	patient, avatar, känna, klient, tvilling, ai, digital, psykolog, risk, kontroll	patient, avatar, feel, client, twin, ai, digital, psychologist, risk, control
3	Relational Loss & the Limits of Simulated Contact	22.9%	människa, möta, mänsklig, ai, patient, kontakt, relation, psykolog, känna, verklig	human, meet, humane, ai, patient, contact, relationship, psychologist, feel, real
4	General Utility & Risk Appraisal	21.2%	ai, patient, använda, svår, behandling, risk, psykolog, känna, viss, positiv	ai, patient, use, difficult, treatment, risk, psychologist, feel, certain, positive

#### 3.2.1 Theme 1: training utility & skill rehearsal

The first topic captured an enthusiastic, utility-oriented view of AI as an instrument for clinical training, and it was overwhelmingly a Virtual Patients response (52.7% mean topic weight across 768 Virtual Patients documents versus 10.0% across 783 Digital Twin documents). High-loading terms (*education*, *role-play*, *training, opportunity*, and *great)* frame simulated patients as a low-stakes environment for rehearsing methods and interview skills, particularly for students and early-career psychologists. One psychologist noted that Virtual Patients “*... could certainly be valuable for psychology students, PTP psychologists, but also licensed psychologists who are faced with scenarios that occur less frequently in the workplace.*”

Responses loading on this topic were more affirmatively framed than those loading on any Digital Twin anchored topic. It was the only topic whose ten highest-probability terms included the emphatic *jättebra* (‘great’), which is consistent with psychologists separating AI as a training tool from AI as a substitute for the clinician. Even within this most positive topic, endorsements typically carried their own caveats. One psychologist cautioned that a simulated patient “*will never be entirely like a real human being*” and might convey stereotypical images of different patient types. Another psychologist called the prospect “*worth its weight in gold*,” because “*psychology is a field where you train a skill at the same time as you practice it for real, and often without feedback*.”

#### 3.2.2 Theme 2: conditional acceptance & clinical authorship

The second topic was the dominant Digital Twin response (40.0% mean topic weight across 783 Digital Twin documents versus 8.9% across 768 Virtual Patients documents) and reflected conditional, qualified engagement rather than outright rejection. The high-loading terms *twin*, *avatar*, *digital*, *risk*, and *control* show psychologists working out three conditions under which a between-session avatar might be acceptable. These were transparency about when the client is and is not communicating with the psychologist, control over what the system says in their name, and oversight of its outputs. As one psychologist put it: “*I feel strong resistance to this. I want to be able to control what ‘I' say to my client, and that feels difficult to achieve through AI.*” Another reflected on the importance of being transparent about “*when it is me and when it is my digital twin,*” noting the risk that a twin “*only confirms the patient instead of challenging them in a helpful way.”*

Acceptance was conditional and centered on authorship. Several psychologists were open to an assistive avatar while explicitly refusing a digital twin of themselves, and others rejected the idea outright. One psychologist called it dangerous, further noting that they *“... cannot take responsibility for what my ‘digital twin’ does, what advice they give or convey to patients, and therefore I cannot vouch that they are ethical. I see risks where my ‘digital twin’ doesn't help the treatment at all or makes a patient upset, and then I, as the therapist, end up in a strange situation where I have to answer for something I haven't done but still bear the responsibility for. Plus, it creates a strange pseudo-relationship between me and the client.”*

#### 3.2.3 Theme 3: relational loss & the limits of simulated contact

The third topic leaned toward the Digital Twin scenario but was substantially present in both (26.3% mean topic weight across 783 Digital Twin documents versus 19.5% across 768 Virtual Patients documents), expressing the relational cost of substituting AI for human contact in either role. High-loading terms like human, meet, contact, and relation foreground what psychologists regarded as irreplaceable, and the exemplar responses came from both scenarios. Reacting to the Virtual Patients vignette, one psychologist wrote that “*we work with people: meeting people is not the same thing as meeting an AI*,” and another noted that simulation “*does not replace group exercises among real people... situations where you feel the patient's anxiety in the room [and] carry responsibility for releasing, or not releasing, a suicidal patient.”* The same relational vocabulary was used against both a therapist surrogate and a training simulation, showing that relational concerns occurred in both of the applications examined.

Where the second topic negotiated conditions for the avatar, this topic questioned the premise, treating the therapeutic relationship itself as the mechanism placed at risk. Psychologists also raised reduced access to conventional talking therapies as a risk. One wrote that they *“believe we have already lost something in that, for example, both children and adolescents no longer have access to talk therapy, but instead receive psychoeducation largely digitally or via videos. The same applies to guardians who get information through psychoeducational videos about emotion regulation, ADHD, or anxiety, but there no longer seems to be room to ask questions and explore their thoughts together with their child and a practitioner.”*

#### 3.2.4 Theme 4: general utility & risk appraisal

The fourth topic was distributed across both scenarios (23.6% mean topic weight across 783 Digital Twin documents versus 18.8% across 768 Virtual Patients documents) and captured a deliberative weighing of circumscribed utility against risk, with high-loading terms such as *use*, *treatment*, *risk*, and *difficult*. Rather than endorsing or rejecting, responses in this topic reasoned about the conditions and side effects of use. One psychologist, for example, saw value in support between sessions early in treatment while noting that “*there is a purpose in not having the psychologist's support all the time... that is how we build independence... and reduce dependence on one's psychologist*,” locating the dependency concern raised elsewhere in the literature within a broader cost–benefit appraisal. Another weighed that AI-generated material “*might lead to increased insight... or it might lead to entirely mistaken notions of how patients behave.”*

Taken with the first topic, this suggests that even the better-received Virtual Patients scenario attracted both enthusiasm for the amount of practice it could provide and doubt about ecological validity, with the fidelity caveats voiced inside the training topic itself and in the cross-cutting relational topic. Another psychologist, responding within the training topic itself, noted that the value of Virtual Patients would lie in honing skills that require repetitive practice with therapeutic methods, but that they *“should of course not completely replace real people. As long as the virtual patients can act realistically enough, it can be useful. But there are obviously many aspects that are left out, such as body language and nuances in speech, and it is difficult to get real-time feedback as things look right now.”*

## 4 Discussion

This study contrasted how a small sample of patients experienced an AI assistant they had actually used against how a large sample of psychologists anticipated future AI applications they had not yet experienced. Across the patient interviews, four themes emerged, spanning analytic engagement, deeper self-understanding, a persistent comparison with a human therapist bound up with stigma, and a shift toward private, on-demand contact.

### 4.1 Patient experience of an AI assistant

The most clinically important pattern was the close link between on-demand access, privacy and stigma avoidance. For a population whose help-seeking is constrained by social-evaluative fear, the value of the AI lay not only in its constant availability but in the fact that no one else could see them using it. One participant described not having told friends about working with an AI therapist. This reframes the “*anytime, anywhere*” advantage emphasized in recent generative-AI trials (Heinz et al., 2025; Hlynsson et al., 2026) in specifically interpersonal terms for social anxiety disorder, and aligns with evidence that help-seeking self-stigma can push patients toward AI-mediated alternatives (Békés and Aafjes-van Doorn, 2026; Hoffman et al., 2024).

At the same time, patients kept comparing the AI with a human therapist (Theme 3). They valued its patience and encouragement but still described something missing in the relationship. One participant noted that the AI felt “*cast in a mold*” and that a human offers a different kind of interaction. This is consistent with theoretical accounts of the therapeutic alliance as a fundamentally human bond (Wampold and Flückiger, 2023), with meta-analytic evidence that the alliance robustly predicts treatment outcome (Flückiger et al., 2018), and with the relational concerns clinicians themselves raise about AI in therapy (Mackey, 2026).

Across the four themes, patients used the AI as an active analytical tool, found it easier to disclose difficult feelings, stayed aware of its limits as a non-human partner, and valued reaching it outside conventional hours. Themes 1 and 2 describe what the format made possible. Theme 3 points to a tension between being understood and being answered by an algorithm. Theme 4, on our reading, suggests that patients experienced treatment as continuously available rather than as a series of scheduled encounters. Because the AI delivered the treatment itself, this was not support between sessions with a human therapist; the present data do not establish what alternative care each participant would otherwise have received. These accounts inform questions about how AI might be positioned within care pathways.

### 4.2 Psychologist attitudes toward prospective applications

The psychologist analysis produced two scenario-anchored topics. These were training utility for Virtual Patients, and authorship-centered conditional acceptance for the Digital Twin. Two further topics cut across both scenarios: a reservation about the loss of genuine human contact, and a general appraisal weighing utility against risk. The sharpest contrast with the patient data lies here. Patients valued being able to reach the AI whenever they needed it, whereas psychologists read constant availability as a risk of dependency (cf. Mackey, 2026). Increased access is not a neutral good when viewed from the provider side. One reading of this contrast, which the present corpus does not itself evidence, is that what a patient experiences as availability may translate for the psychologist into demands on time and attention within an already structured working day. We return to this contrast below.

### 4.3 The role assigned to AI

Read on their own terms, the psychologist themes suggest that openness to generative AI depended less on the technology's capability than on the role it was assigned. Affirmative framing concentrated where AI stands in for the patient in low-stakes rehearsal (Theme 1), and fell away once AI stood in for the psychologist (Themes 2 and 3). The Digital Twin reservations were not doubts about competence but questions of authorship and relational integrity. Psychologists wanted control over what they would say through a digital twin, and explicit labeling of when the client was not speaking with the psychologist directly. They treated this as a matter of responsibility, locating the risk in the therapeutic relationship rather than in any deficiency of the model (Flückiger et al., 2018; Wampold and Flückiger, 2023).

While the Digital Twin vignette is hypothetical for these respondents, it is not speculative as a technology. Frameworks for building digital twins of psychological counselors are under active development, and analyses of this work warn that the twin metaphor itself invites misplaced trust and over-disclosure, and call for empirical evidence on the attitudes such labels elicit (Annoni et al., 2026). The conditions psychologists raised unprompted (i.e., consent, explicit labeling, and preserved responsibility) mirror the consent, transparency, and authentic-presence conditions proposed for personalized digital duplicates in the ethics literature (Danaher and Nyholm, 2025).

The training scenario also raised concerns about fidelity to real clinical encounters. Virtual-patient applications are being explored in medical and mental health training (Borg et al., 2025; Reger et al., 2021). Neither scenario received unqualified endorsement: reservations concerned fidelity in simulation and responsibility and the therapeutic relationship in surrogacy. This pattern is consistent with reports of clinicians accepting AI as an adjunct while resisting replacement (Békés and Aafjes-van Doorn, 2026; Cecil et al., 2025; Mackey, 2026; Rahsepar Meadi et al., 2025; Wagner and Schwind, 2025). The apparent preference for training applications is a hypothesis about acceptability, given the descriptive analysis and different scenario vocabularies.

### 4.4 Convergence and divergence between the two perspectives

Setting the two corpora side by side answers the study's third question, with one caveat. Patients describe a real tool that delivered their treatment, whereas psychologists reason about applications they have not used, so the two accounts are not measurements of the same object and the comparison below is interpretive. The clearest convergence is relational. The patients' human-AI comparison (Theme 3), in which the AI was experienced as formulaic relative to a human therapist, maps directly onto the psychologists' relational theme, which pooled the loss of human contact and the limits of simulated encounters across both scenarios. From opposite standpoints, both groups located the technology's ceiling in the relationship it cannot provide.

The comparison also concerns different roles assigned to AI. Patients valued the availability and privacy of the treatment they had used. Psychologists raised concerns about authorship, oversight and dependency in the hypothetical between-session Digital Twin scenario. These accounts concern different applications and reference points, so they do not establish opposing judgments about the same treatment.

### 4.5 Limitations

The study has limitations. Most importantly, the patient findings rest on eleven participants and short interview segments. The themes are exploratory and hypothesis-generating, and require confirmation in larger samples. Topic models are bag-of-words methods blind to negation and context, so interpretive weight rests on the close reading of high-loading segments rather than on the statistical model alone. A second and separately documented risk is that on short, low-diversity open-ended survey responses, topic modeling yields poorly differentiated topics (Maslej et al., 2025). The present psychologist corpus likely fared better because the two vignettes supplied distinct vocabularies and because the lemma-aware pipeline and manual exemplar validation constrained interpretation. As noted in the Introduction, Southwick et al. (2024) applied LDA to open-ended responses from both patients and therapists after a randomized trial, but in that study both groups had been exposed to the same intervention, which is not the case here. Although interviewer turns were removed, the patient turns themselves included non-substantive material (e.g., logistical exchanges and non-answers) that topic models cannot distinguish from content. In the psychologist free-text data, part of the lexical separation between scenarios is mechanical because the vignettes themselves introduce distinct vocabularies (e.g., “*twin*” and “*avatar*” versus “*training*” and “*role-play*”); the substantive finding is therefore the within-scenario split into engagement and reservation rather than the between-scenario separation itself. In addition, both scenarios were presented to each psychologist, and this within-person pairing is not modeled by the LDA. Prevalence stability under participant weighting and theme distribution across all participants partly offset, but do not remove, these limitations.

The two corpora also differ in clinical scope. The patient data are specific to social anxiety disorder, whereas the psychologist vignettes addressed clinical AI generally, so group differences are confounded with domain. Self-selection affects both samples. Requiring telephone interviews excluded three patients who preferred a text-only interview, which may have limited the range of experiences represented. The interviewed patients had consented to AI-delivered treatment and then volunteered for interviews, so the corpus likely over-represents people already open to AI, and the psychologists were recruited through a single professional association's channels, with respondents holding strong views on AI plausibly over-represented. Both corpora come from one country, and Sweden combines high digital literacy, publicly financed care, and a long tradition of internet-delivered treatment, so the reasoning documented here need not generalize to other healthcare systems. Finally, the analyses were run on Swedish text while all quotations are presented in English translation, and translation is itself an interpretive step.

## 5 Conclusions

Patients valued AI-delivered treatment as accessible and private, while psychologists were more receptive to simulated patients for training than to an avatar acting on their behalf. These descriptive findings concern different populations, applications and forms of exposure. They should be weighed against documented safety concerns (Moore et al., 2025) and limits on replacing human providers (Schuster et al., 2026). Future studies should compare patient and psychologist perspectives on the same specified application after actual use, recruit larger and more varied patient samples, and model paired scenario responses within psychologists.

## Ethical statement

The interview study was covered by approval from the Swedish Ethical Review Authority (Dnr: 2025-01603-02). The Authority determined that the psychologist survey containing the two scenarios fell outside the scope of mandatory ethical review (Dnr: 2025-00064-01).

## Declaration of Generative AI and AI-assisted technologies in the writing process

During manuscript preparation and revision, the authors used Claude Fable 5.1 (Anthropic, via Claude Code) and Astra 6 (OpenAI, via Codex) for language editing and critical feedback. The authors reviewed and edited the content and take responsibility for the published article.

## Funding

This research did not receive any specific grant from funding agencies in the public, commercial, or not-for-profit sectors.

## CRediT authorship contribution statement

**Jón Ingi Hlynsson:** Writing – original draft, Writing – review & editing, Methodology, Software, Formal analysis, Data curation, Validation, Visualization. **Kristofer Vernmark:** Writing – review & editing, Conceptualization, Methodology, Investigation, Resources. **Catharina Lutovsky:** Writing – review & editing, Investigation, Data curation. **Gerhard Andersson:** Writing – review & editing, Conceptualization, Methodology, Resources, Supervision. **Per Carlbring:** Writing – review & editing, Conceptualization, Methodology, Validation, Resources, Supervision, Project administration.

## Declaration of competing interest

Per Carlbring and Gerhard Andersson are Editors of Internet Interventions. They had no involvement in, or access to, the editorial process or peer review of this manuscript, which was handled by an independent editor. Both studies were run on the Iterapi platform, which the authors' research group develops and maintains (Vlaescu et al., 2026). The authors declare no other competing interests.

## Data Availability

The analysis code and the analytic workflow are available in the supplementary material. The interview transcripts and free-text survey responses cannot be shared publicly, as they could contain indirectly identifying information; de-identified data are available from the corresponding author on reasonable request.
