# Supplementary material for: Patient experience and psychologists' attitudes toward AI in psychological treatment: A topic-modeling study
Source: Internet Interv. 2026 Sep 18;46:100999. doi: 10.1016/j.invent.2026.100999 (PMC13639969; doi:10.1016/j.invent.2026.100999)
Supplement: Supplementary file 1 [file mmc1.zip › supplementary_material_html_FINAL.html]

Supplementary Material


Code 

- Show All Code
- Hide All Code

# Supplementary Material

### Patient experience and psychologists’ attitudes toward AI in psychological treatment: A topic-modeling study

#### Hlynsson et al.

#### 2026-09-16

# Overview

This supplement contains the complete analysis code for both topic
models reported in the manuscript, together with the model-selection
evidence (coherence by *k*, lexical distinctness, seed
stability), the document-flow accounting for both corpora, the
robustness checks (participant weighting, single-participant share), a
short-text benchmark model, and a check that every quotation presented
in the manuscript loads on the topic under which it appears. Section S7
reports a post-submission audit in which both models were refitted from
the raw data and every quotation was rechecked.

Reproducibility parameters used throughout: Latent Dirichlet
Allocation via `textmineR::FitLdaModel`, *k* = 4,
2,000 Gibbs iterations, α = 0.1, β = 0.5; document–term matrices built
with n-grams of length 1–3 added alongside their constituent unigrams;
terms retained if they appear in ≥ 2 documents; a single lemma-aware
stop list applied identically to both corpora; topic prevalence
operationalised as the mean posterior document–topic probability with
documents weighted equally. All models are estimated with
`seed = 12345`.

Tables S9 to S12 were added after Tables S1 to S8 had been numbered
and referenced, so they appear out of numerical sequence in the
document. Each is referenced from the section it belongs to.

**Provenance.** This document was knitted with
`run_models = TRUE`. Every table and figure below was
computed from the raw data during this knit.

# S1. Software

```
sessionInfo()
```

```
## R version 4.3.3 (2024-02-29 ucrt)
## Platform: x86_64-w64-mingw32/x64 (64-bit)
## Running under: Windows 11 x64 (build 26200)
## 
## Matrix products: default
## 
## 
## locale:
## [1] LC_COLLATE=English_Sweden.utf8  LC_CTYPE=English_Sweden.utf8   
## [3] LC_MONETARY=English_Sweden.utf8 LC_NUMERIC=C                   
## [5] LC_TIME=English_Sweden.utf8    
## 
## time zone: Europe/Stockholm
## tzcode source: internal
## 
## attached base packages:
## [1] stats     graphics  grDevices utils     datasets  methods   base     
## 
## loaded via a namespace (and not attached):
##  [1] digest_0.6.37     R6_2.6.1          fastmap_1.2.0     xfun_0.60        
##  [5] cachem_1.1.0      knitr_1.51        htmltools_0.5.8.1 rmarkdown_2.28   
##  [9] lifecycle_1.0.4   cli_3.6.4         sass_0.4.9        jquerylib_0.1.4  
## [13] compiler_4.3.3    rstudioapi_0.17.0 tools_4.3.3       evaluate_1.0.3   
## [17] bslib_0.8.0       yaml_2.3.10       rlang_1.1.5       jsonlite_1.9.1
```

```
cat(if (isTRUE(params$refit_from_scratch))
  "The cache was cleared before this knit, so every model was fitted afresh.\n" else "")
```

```
## The cache was cleared before this knit, so every model was fitted afresh.
```

# S2. Shared preprocessing pipeline

Both corpora pass through the identical pipeline: raw-text
abbreviation scrubbing, UDPipe lemmatisation (Swedish–Talbanken),
lemma-family normalisation, a shared stop list, and a document–term
matrix with 1–3-grams and a document-frequency floor of 2. Documents
that lose all content words at any stage are counted and excluded.

The patient corpus consists of **patient turns only**.
Interviewer turns are removed at read-in by
`filter(group == "patient")`, before any preprocessing, so no
interviewer speech enters the document–term matrix. The count of
excluded interviewer turns is reported in Table S1.

```
library(readxl); library(dplyr); library(stringr)
library(udpipe); library(BTM); library(textmineR); library(Matrix)
library(stopwords)

SEED          <- 12345
ALT_SEEDS     <- c(2026, 42)
PATIENT_XLSX  <- "clean_transcripts.xlsx"
SCENARIO_XLSX <- "AI-scenarion-dataset-edited.xlsx"
EXEMPLAR_MIN  <- 15   # minimum content tokens for exemplar extraction

if (!exists("udmodel")) {
  dl <- tryCatch(udpipe_load_model("swedish-talbanken-ud-2.5-191206.udpipe"),
                 error = function(e) NULL)
  if (is.null(dl)) { m <- udpipe_download_model(language = "swedish")
                     dl <- udpipe_load_model(m$file_model) }
  udmodel <- dl
}

# Raw-text scrub BEFORE lemmatisation: abbreviations that otherwise shed
# n-gram debris (t.ex., dvs, bl.a., m.m., o.s.v.)
scrub_raw <- function(x) {
  x <- gsub("\\bt\\.?\\s*ex\\.?\\b", " ", x, ignore.case = TRUE)
  x <- gsub("\\b(d\\.?v\\.?s|bl\\.?a|m\\.?m|o\\.?s\\.?v)\\.?\\b", " ",
            x, ignore.case = TRUE)
  x
}

# Lemma-family normalisation AFTER lemmatisation, so that no truncated or
# duplicated lemma variants (e.g., avat/avatare, möt/möta) enter the DTM.
normalise_lemmas <- function(x) {
  x %>%
    str_replace_all("\\b(kände|känns|känner|kännas)\\b", "känna") %>%
    str_replace_all("\\bförståelse\\b", "förstå") %>%
    str_replace_all("(?i)\\b(ai_n|ai:n|ain|ai:in)\\b", "ai") %>%
    str_replace_all("\\bavat(ar|are|arer|aren|arerna)?\\b", "avatar") %>%
    str_replace_all("\\bmöt(a|e|en|et|as)?\\b", "möta") %>%
    str_replace_all("\\btvilling(en|ar|arna)?\\b", "tvilling")
}

# Shared lemma-aware stop list
stop_vec_sv <- stopwords::stopwords("sv", source = "snowball")
conversational_noise <- c(
  "mm","aa","mhm","aha","alltså","liksom","typ","ba","nää",
  "lite","väldigt","ganska","mycket","mer","bara","nog","väl","ju","så",
  "just","precis","exakt","riktigt","verkligen","egentligen","faktiskt",
  "sådär","såhär","ändå","annat","olika","helt","alls","oftast","ofta",
  "otroligt","längre","aldrig","hela","in",
  "ja","jo","nej","nä","okej","absolut","visst",
  "säga","prata","göra","få","kunna","skola","vilja","komma",
  "ge","ta","bli","finnas","se","gå","fortsätta","behöva",
  "kanske","veta","tro","tycka","tänka","uppleva",
  "sak","ting","grej","sätt","vis","del","gång","dag","tid",
  "person","man","en","ett","någonting","något","exempel","problem",
  "rätt","fel","stor","liten","sen","då","nu","här","där","hit","dit",
  "innan","efter")
lemma_noise <- c("väldig","riktig","sån","sådan","också","all","annan",
  "ibland","fall","faktisk","två","kring","börja","början",
  "kännas","stund","minut","vecka","olik","otrolig",
  "t","ex","t_ex","tex","dvs","bl_a","bla","m_m")
evaluative <- c("bra","god","dålig")
full_stop_list <- unique(c(stop_vec_sv, conversational_noise,
                           lemma_noise, evaluative))

lemmatize_col <- function(df, text_col, id_col) {
  ann <- udpipe_annotate(udmodel, x = scrub_raw(tolower(df[[text_col]])),
                         doc_id = as.character(df[[id_col]])) %>% as.data.frame()
  ann %>%
    filter(upos != "PUNCT", !is.na(lemma)) %>%
    mutate(lemma = normalise_lemmas(lemma)) %>%
    group_by(doc_id) %>%
    summarise(lemmatized_text = paste(lemma, collapse = " "), .groups = "drop")
}

build_dtm <- function(texts, names) {
  d <- CreateDtm(doc_vec = texts, doc_names = names,
                 ngram_window = c(1, 3),   # n-grams added alongside unigrams
                 stopword_vec = full_stop_list, lower = TRUE,
                 remove_punctuation = TRUE, remove_numbers = TRUE)
  d[, colSums(d > 0) >= 2]
}

# Top-10 terms of a fitted textmineR model, as a list of character vectors
top_terms_list <- function(model, M = 10) {
  tt <- GetTopTerms(model$phi, M = M)
  lapply(seq_len(ncol(tt)), function(j) as.character(tt[, j]))
}

# Pairwise shared top-10 terms and cosine similarity between topic-term
# distributions. Used for BOTH corpora (Tables S7 and S9).
distinctness <- function(model, M = 10) {
  phi <- model$phi
  tl  <- top_terms_list(model, M)
  k   <- length(tl)
  nm  <- paste0("t_", seq_len(k))
  shared <- matrix(NA_integer_, k, k, dimnames = list(nm, nm))
  for (i in seq_len(k)) for (j in seq_len(k)) if (i != j)
    shared[i, j] <- length(intersect(tl[[i]], tl[[j]]))
  flr <- names(which(table(unlist(tl)) >= 3))   # corpus-floor terms
  shared_nf <- matrix(NA_integer_, k, k, dimnames = list(nm, nm))
  for (i in seq_len(k)) for (j in seq_len(k)) if (i != j)
    shared_nf[i, j] <- length(setdiff(intersect(tl[[i]], tl[[j]]), flr))
  nrm  <- sqrt(rowSums(phi^2))
  cosm <- (phi %*% t(phi)) / outer(nrm, nrm)
  diag(cosm) <- NA
  dimnames(cosm) <- list(nm, nm)
  list(shared = shared, shared_nofloor = shared_nf,
       cosine = round(cosm, 2), floor_terms = flr, terms = tl)
}

# Seed-stability check (applied to BOTH corpora below): refit at alternative
# seeds, greedy-match topics between runs by cosine similarity of their term
# distributions, and report the top-10 term overlap of matched topics.
seed_stability <- function(dtm, ref_model, seeds = ALT_SEEDS) {
  phi <- ref_model$phi; tt <- GetTopTerms(phi, M = 10)
  out <- sapply(seeds, function(s) {
    set.seed(s)
    m2 <- FitLdaModel(dtm, k = nrow(phi), iterations = 2000,
                      alpha = 0.1, beta = 0.5)
    tt2 <- GetTopTerms(m2$phi, M = 10)
    n1 <- sqrt(rowSums(phi^2)); n2 <- sqrt(rowSums(m2$phi^2))
    cm <- (phi %*% t(m2$phi)) / outer(n1, n2)
    used <- integer(0); ov <- numeric(nrow(phi))
    for (i in seq_len(nrow(phi))) {
      j <- setdiff(order(cm[i, ], decreasing = TRUE), used)[1]
      used <- c(used, j)
      ov[i] <- length(intersect(tt[, i], tt2[, j])) / 10
    }
    ov
  })
  dimnames(out) <- list(paste0("t_", seq_len(nrow(out))), paste0("seed_", seeds))
  out
}

# Exemplar extraction. Returns identifiers and loadings; the text column is
# included only when print_exemplar_text = TRUE.
exemplars <- function(model, dtm, meta, id_col, text_col, topic_labels,
                      n = 3, min_tokens = EXEMPLAR_MIN, show_text = SHOW_TEXT) {
  tk   <- Matrix::rowSums(dtm)
  long <- which(tk >= min_tokens)
  out  <- lapply(seq_len(ncol(model$theta)), function(k) {
    cand <- long[order(model$theta[long, k], decreasing = TRUE)]
    idx  <- head(cand, n)
    d <- data.frame(
      topic    = paste0("t_", k),
      label    = topic_labels[k],
      participant = as.character(meta[[id_col]][idx]),
      segment  = as.character(meta$seg_id[idx]),
      theta    = round(model$theta[idx, k], 2),
      tokens   = as.integer(tk[idx]),
      stringsAsFactors = FALSE)
    if (show_text)
      d$text <- substring(meta[[text_col]][idx], 1, 300)
    d
  })
  do.call(rbind, out)
}
```

# S3. Patient corpus

## S3.1 Analysis code

```
pat_all <- read_excel(PATIENT_XLSX)

# Interviewer turns are removed here, before any preprocessing.
pat_raw <- pat_all %>%
  filter(group == "patient") %>%
  mutate(seg_id = row_number())
n_interviewer_turns <- nrow(pat_all) - nrow(pat_raw)

blank_raw_pat <- sum(str_trim(replace(pat_raw$text, is.na(pat_raw$text), "")) == "")
pat_lem <- lemmatize_col(pat_raw, "text", "seg_id")
pat_raw$lem <- pat_lem$lemmatized_text[match(as.character(pat_raw$seg_id),
                                             pat_lem$doc_id)]
pat_raw$lem[is.na(pat_raw$lem)] <- ""
pat_keep <- pat_raw %>% filter(str_trim(lem) != "")
n_emptied_pat <- (nrow(pat_raw) - blank_raw_pat) - nrow(pat_keep)

dtm_pat  <- build_dtm(pat_keep$lem, as.character(pat_keep$seg_id))
tok_pat  <- Matrix::rowSums(dtm_pat)
n_pruned_pat <- sum(tok_pat == 0)
dtm_pat  <- dtm_pat[tok_pat > 0, ]            # drop docs emptied by pruning
pat_keep <- pat_keep[tok_pat > 0, ]

lda_pat <- cached("lda_pat", {
  set.seed(SEED)
  FitLdaModel(dtm_pat, k = 4, iterations = 2000,
              alpha = 0.1, beta = 0.5, calc_coherence = TRUE)
})

prev_pat_doc <- round(100 * colMeans(lda_pat$theta), 1)   # document-weighted

# Participant-weighted prevalence (robustness)
prev_pat_part <- as.data.frame(lda_pat$theta) %>%
  mutate(pid = pat_keep$doc_ID) %>% group_by(pid) %>%
  summarise(across(everything(), mean), .groups = "drop") %>%
  select(-pid) %>% colMeans()
prev_pat_part <- round(100 * prev_pat_part, 1)

# Largest single-participant share of each topic's mass
share_pat <- sapply(1:4, function(k) {
  mass <- tapply(lda_pat$theta[, k], pat_keep$doc_ID, sum)
  round(100 * max(mass) / sum(mass), 1)
})

# Coherence by k (Table S3)
coh_pat <- cached("coh_pat", sapply(2:15, function(kk) {
  set.seed(SEED)
  mean(FitLdaModel(dtm_pat, k = kk, iterations = 2000, alpha = 0.1,
                   beta = 0.5, calc_coherence = TRUE)$coherence)
}))

# Seed stability (Table S3 text, S4.6)
seed_pat <- cached("seed_pat", seed_stability(dtm_pat, lda_pat))

# Lexical distinctness (Table S9)
dist_pat <- distinctness(lda_pat)

# Biterm benchmark for short texts (Table S10; Yan et al., 2013)
pat_tok_long <- {
  ann <- udpipe_annotate(udmodel, x = scrub_raw(tolower(pat_keep$text)),
                         doc_id = as.character(pat_keep$seg_id)) %>%
    as.data.frame()
  ann %>% filter(upos != "PUNCT", !is.na(lemma)) %>%
    mutate(token = normalise_lemmas(lemma)) %>%
    filter(!token %in% full_stop_list, !str_detect(token, "^[0-9]+$"),
           nchar(token) > 1) %>% select(doc_id, token)
}
btm_pat <- cached("btm_pat", {
  set.seed(SEED)
  BTM(data = pat_tok_long, k = 4, beta = 0.01, iter = 2000, window = 15)
})

# Exemplars, length-floored (text withheld unless print_exemplar_text = TRUE)
ex_pat <- exemplars(lda_pat, dtm_pat, pat_keep, "doc_ID", "text", PAT_LABELS)
```

## S3.2 Document flow (Table S1)

Table S1. Patient corpus document flow. Interviewer turns are
excluded before preprocessing, so every analyzable segment is patient
speech.

| Stage | n |
| --- | --- |
| Interviewer turns excluded at read-in | 666 |
| Patient turns collected (11 participants) | 443 |
| Blank at source | 0 |
| Emptied by lemmatisation/stop list | 0 |
| Zeroed by DTM pruning (document frequency < 2) | 53 |
| Analyzable patient segments | 390 |

## S3.3 Final model (Table S2)

Table S2. Patient model (k = 4, seed 12345): topics in model
order with their manuscript theme labels, prevalence under both
weightings, the largest single-participant contribution to each topic,
and top terms.

| Topic | Manuscript theme | Prevalence (doc-weighted) | Prevalence (participant-weighted) | Top single-participant share | Top 10 terms |
| --- | --- | --- | --- | --- | --- |
| t\_1 | 1 Analytic Engagement & Situational Insight | 25.1% | 25.5% | 17.6% | ai, direkt, övning, terapeut, jobb, känd, jobba, prova, nå, situation |
| t\_2 | 4 On-Demand Access & Engagement Logistics | 27.6% | 28.1% | 16.8% | känna, skriva, gärna, samtal, nästan, svår, fråga, lägga, telefon, jobbig |
| t\_3 | 2 Deep Clinical Processing & Self-Understanding | 22.3% | 21.8% | 19% | situation, social, ångest, förstå, social\_ångest, känna, hjälp, fråga, dels, tidig |
| t\_4 | 3 Relational Authenticity, Stigma & Human-AI Comparison | 25% | 24.6% | 17.5% | känna, ai, människa, svar, terapeut, förstå, hjälpa, använda, insikt, fråga |

## S3.4 Coherence by *k* (Table S3, Figure S1)

Table S3. Patient corpus: mean probabilistic coherence by k.
The trajectory does not sharply favour any single k; k = 4 was retained
on interpretability and lexical distinctness rather than on coherence,
as higher k fragmented core themes (Table S8).

| k | mean\_coherence |
| --- | --- |
| 2 | 0.0585 |
| 3 | 0.0703 |
| 4 | 0.0945 |
| 5 | 0.0604 |
| 6 | 0.0785 |
| 7 | 0.0958 |
| 8 | 0.0760 |
| 9 | 0.0857 |
| 10 | 0.0931 |
| 11 | 0.0778 |
| 12 | 0.0944 |
| 13 | 0.0840 |
| 14 | 0.0930 |
| 15 | 0.0858 |

## S3.5 Lexical distinctness (Table S9)

The patient model is reported here on the same distinctness criteria
applied to the psychologist model in Table S7, so that the two corpora
are described on equal terms.

Table S9. Patient topics: pairwise shared top-10
terms.

| topic | t\_1 | t\_2 | t\_3 | t\_4 |
| --- | --- | --- | --- | --- |
| t\_1 | NA | 0 | 1 | 2 |
| t\_2 | 0 | NA | 2 | 2 |
| t\_3 | 1 | 2 | NA | 3 |
| t\_4 | 2 | 2 | 3 | NA |

Table S9 (continued). Shared top-10 terms excluding
corpus-floor terms, defined as terms appearing in at least three topics’
top-10 lists (fråga, känna).

| topic | t\_1 | t\_2 | t\_3 | t\_4 |
| --- | --- | --- | --- | --- |
| t\_1 | NA | 0 | 1 | 2 |
| t\_2 | 0 | NA | 0 | 0 |
| t\_3 | 1 | 0 | NA | 1 |
| t\_4 | 2 | 0 | 1 | NA |

Table S9 (continued). Cosine similarity between patient
topic-term distributions.

| topic | t\_1 | t\_2 | t\_3 | t\_4 |
| --- | --- | --- | --- | --- |
| t\_1 | NA | 0.35 | 0.42 | 0.46 |
| t\_2 | 0.35 | NA | 0.41 | 0.62 |
| t\_3 | 0.42 | 0.41 | NA | 0.43 |
| t\_4 | 0.46 | 0.62 | 0.43 | NA |

## S3.6 Short-text benchmark (Table S10)

Because the patient corpus consists of short segments, the four-topic
LDA solution was benchmarked against a biterm topic model, which is
designed for short texts and models term co-occurrence at the corpus
level rather than within documents (Yan et al., 2013). The biterm model
was fitted on unigram lemmas only, whereas the LDA document–term matrix
also contains 2- and 3-grams, so exact term overlap understates
agreement between the two solutions. The table reports, for each LDA
topic, the biterm topic it matches most closely and the number of top-10
terms the two share.

Table S10. Biterm topic model benchmark (k = 4, seed 12345)
against the patient LDA solution, greedy-matched by top-10 term
overlap.

| LDA topic | Manuscript theme | Matched BTM topic | Shared top-10 terms | BTM top terms |
| --- | --- | --- | --- | --- |
| t\_1 | 1 Analytic Engagement & Situational Insight | b\_1 | 4 | känna, förstå, prova, irriterad, gran, försöka, nå, direkt, beskriva, situation |
| t\_2 | 4 On-Demand Access & Engagement Logistics | b\_3 | 3 | känna, skriva, fråga, situation, fungera, svar, istället, lika, övning, kväll |
| t\_3 | 2 Deep Clinical Processing & Self-Understanding | b\_2 | 6 | känna, ångest, fråga, ai, situation, social, förstå, svar, hel, hjälpa |
| t\_4 | 3 Relational Authenticity, Stigma & Human-AI Comparison | b\_4 | 4 | känna, förstå, situation, övning, social, ai, människa, skriva, hel, ångest |

## S3.7 Exemplars

Patient exemplars: the three highest-loading documents per
topic subject to a floor of 15 content tokens. Participant text is
withheld by default; set print\_exemplar\_text = TRUE to include it in a
local, unshared knit.

| topic | label | participant | segment | theta | tokens |
| --- | --- | --- | --- | --- | --- |
| t\_1 | 1 Analytic Engagement & Situational Insight | p\_1030 | 35 | 0.99 | 43 |
| t\_1 | 1 Analytic Engagement & Situational Insight | p\_1014 | 72 | 0.98 | 19 |
| t\_1 | 1 Analytic Engagement & Situational Insight | p\_1065 | 151 | 0.98 | 17 |
| t\_2 | 4 On-Demand Access & Engagement Logistics | p\_1014 | 57 | 0.99 | 44 |
| t\_2 | 4 On-Demand Access & Engagement Logistics | p\_1030 | 27 | 0.99 | 27 |
| t\_2 | 4 On-Demand Access & Engagement Logistics | p\_1129 | 270 | 0.99 | 23 |
| t\_3 | 2 Deep Clinical Processing & Self-Understanding | p\_1125 | 213 | 0.99 | 29 |
| t\_3 | 2 Deep Clinical Processing & Self-Understanding | p\_1125 | 212 | 0.99 | 25 |
| t\_3 | 2 Deep Clinical Processing & Self-Understanding | p\_1129 | 260 | 0.99 | 25 |
| t\_4 | 3 Relational Authenticity, Stigma & Human-AI Comparison | p\_1014 | 78 | 0.99 | 31 |
| t\_4 | 3 Relational Authenticity, Stigma & Human-AI Comparison | p\_1014 | 53 | 0.99 | 25 |
| t\_4 | 3 Relational Authenticity, Stigma & Human-AI Comparison | p\_1014 | 86 | 0.99 | 24 |

# S4. Psychologist corpus

## S4.1 Analysis code

```
sc_raw <- read_excel(SCENARIO_XLSX)
names(sc_raw)[1:3] <- c("Senario", "ID", "text")
sc_raw <- sc_raw %>% mutate(seg_id = row_number(), Senario = factor(Senario))

blank_raw <- sum(str_trim(replace(sc_raw$text, is.na(sc_raw$text), "")) == "")
sc_lem <- lemmatize_col(sc_raw, "text", "seg_id")
sc_raw$lem <- sc_lem$lemmatized_text[match(as.character(sc_raw$seg_id),
                                           sc_lem$doc_id)]
sc_raw$lem[is.na(sc_raw$lem)] <- ""
sc_keep <- sc_raw %>% filter(str_trim(lem) != "")
# Documents emptied in preprocessing = all lost before DTM pruning, minus the
# source blanks (which are reported on their own row). Never negative.
# n_emptied_sc is derived in the Table S4 chunk from the final totals,
# after all filtering, so it cannot depend on intermediate object states.

dtm_sc  <- build_dtm(sc_keep$lem, as.character(sc_keep$seg_id))
tok0    <- Matrix::rowSums(dtm_sc)
dtm_sc  <- dtm_sc[tok0 > 0, ]; sc_keep <- sc_keep[tok0 > 0, ]

n_by_scenario <- table(sc_keep$Senario)   # analyzable per scenario
n_psychologists <- n_distinct(sc_keep$ID) # psychologists contributing
n_both <- sum(table(sc_keep$ID) == 2)     # contributing to both scenarios

# Coherence by k (Table S5)
coh_sc <- cached("coh_sc", sapply(2:15, function(kk) {
  set.seed(SEED)
  mean(FitLdaModel(dtm_sc, k = kk, iterations = 2000, alpha = 0.1,
                   beta = 0.5, calc_coherence = TRUE)$coherence)
}))

lda_sc <- cached("lda_sc", {
  set.seed(SEED)
  FitLdaModel(dtm_sc, k = 4, iterations = 2000,
              alpha = 0.1, beta = 0.5, calc_coherence = TRUE)
})

th <- lda_sc$theta
prev_sc <- round(100 * colMeans(th), 1)
bs <- do.call(rbind, lapply(levels(sc_keep$Senario), function(s)
  colMeans(th[sc_keep$Senario == s, , drop = FALSE])))
bs <- round(100 * bs, 1); rownames(bs) <- levels(sc_keep$Senario)

# Lexical distinctness (Table S7)
dist_sc <- distinctness(lda_sc)

# Seed stability (S4.6)
seed_sc <- cached("seed_sc", seed_stability(dtm_sc, lda_sc))

# Exemplars, length-floored (text withheld unless print_exemplar_text = TRUE)
ex_sc <- exemplars(lda_sc, dtm_sc, sc_keep, "ID", "text", PSY_LABELS)
```

## S4.2 Document flow (Table S4)

Table S4. Psychologist corpus document flow.

| Stage | n |
| --- | --- |
| Psychologists submitting a response to both scenario prompts | 818 |
| Raw responses (2 per psychologist) | 1636 |
| Blank at source | 2 |
| Emptied of content words in preprocessing | 83 |
| Analyzable documents | 1551 |
| of which Digital Twin (Scenario 1) | 783 |
| of which Virtual Patients (Scenario 2) | 768 |
| Psychologists with at least 1 analyzable document | 808 |
| Psychologists contributing analyzable documents to both scenarios | 743 |

## S4.3 Coherence by *k* (Table S5, Figure S2)

Table S5. Psychologist corpus: mean probabilistic coherence by
k (maximum at k = 10). Probabilistic coherence rewards granular,
near-duplicate topics in short text (Chang et al., 2009), so k was
selected on interpretability and lexical distinctness (Table S7) rather
than by maximising coherence. The retained solution therefore sits below
the coherence maximum by design.

| k | mean\_coherence |
| --- | --- |
| 2 | 0.0554 |
| 3 | 0.0760 |
| 4 | 0.0725 |
| 5 | 0.0691 |
| 6 | 0.0748 |
| 7 | 0.0779 |
| 8 | 0.0715 |
| 9 | 0.0815 |
| 10 | 0.0946 |
| 11 | 0.0850 |
| 12 | 0.0825 |
| 13 | 0.0831 |
| 14 | 0.0888 |
| 15 | 0.0830 |

## S4.4 Final model (Table S6)

Table S6. Psychologist model (k = 4, seed 12345): topics in
model order with manuscript theme labels, corpus prevalence, by-scenario
prevalence, and top terms. Two topics are scenario-anchored; two cut
across scenarios.

| Topic | Manuscript theme | Prevalence | Digital Twin | Virtual Patients | Top 10 terms |
| --- | --- | --- | --- | --- | --- |
| t\_1 | 4 General Utility & Risk Appraisal | 21.2% | 23.6% | 18.8% | ai, patient, använda, svår, behandling, risk, psykolog, känna, viss, positiv |
| t\_2 | 2 Conditional Acceptance & Clinical Authorship | 24.6% | 40% | 8.9% | patient, avatar, känna, klient, tvilling, ai, digital, psykolog, risk, kontroll |
| t\_3 | 3 Relational Loss & the Limits of Simulated Contact | 22.9% | 26.3% | 19.5% | människa, möta, mänsklig, ai, patient, kontakt, relation, psykolog, känna, verklig |
| t\_4 | 1 Training Utility & Skill Rehearsal | 31.2% | 10% | 52.7% | låta, utbildning, positiv, patient, träna, rollspel, jättebra, psykolog, möjlighet, verklig |

## S4.5 Lexical distinctness (Table S7)

Table S7. Psychologist topics: pairwise shared top-10
terms.

| topic | t\_1 | t\_2 | t\_3 | t\_4 |
| --- | --- | --- | --- | --- |
| t\_1 | NA | 5 | 4 | 3 |
| t\_2 | 5 | NA | 4 | 2 |
| t\_3 | 4 | 4 | NA | 3 |
| t\_4 | 3 | 2 | 3 | NA |

Table S7 (continued). Shared top-10 terms excluding
corpus-floor terms (ai, känna, patient, psykolog).

| topic | t\_1 | t\_2 | t\_3 | t\_4 |
| --- | --- | --- | --- | --- |
| t\_1 | NA | 1 | 0 | 1 |
| t\_2 | 1 | NA | 0 | 0 |
| t\_3 | 0 | 0 | NA | 1 |
| t\_4 | 1 | 0 | 1 | NA |

Table S7 (continued). Cosine similarity between psychologist
topic-term distributions.

| topic | t\_1 | t\_2 | t\_3 | t\_4 |
| --- | --- | --- | --- | --- |
| t\_1 | NA | 0.55 | 0.48 | 0.44 |
| t\_2 | 0.55 | NA | 0.45 | 0.35 |
| t\_3 | 0.48 | 0.45 | NA | 0.41 |
| t\_4 | 0.44 | 0.35 | 0.41 | NA |

## S4.6 Seed stability

Both models were refitted at alternative seeds (2026 and 42) and
topics were matched between runs by cosine similarity of their term
distributions. The table reports the mean top-10 term overlap of matched
topics.

Mean top-10 term overlap between matched topics under
alternative seeds.

| Corpus | Seed 2026 | Seed 42 |
| --- | --- | --- |
| Patient (390 short segments) | 52% | 60% |
| Psychologist (1,551 documents) | 88% | 75% |

The psychologist solution re-emerges under both alternative seeds,
indicating that its four-topic structure is not an artefact of the seed.
The patient model, fitted on a far smaller corpus, is only moderately
seed-stable. Its themes therefore rest additionally on the
exemplar-based validation (S3.7), the short-text benchmark (Table S10),
and the quotation check (Table S11) rather than on the topic solution
alone. This limitation is stated in the manuscript.

## S4.7 Exemplars

Psychologist exemplars: the three highest-loading documents per
topic subject to a floor of 15 content tokens. Respondent text is
withheld by default; set print\_exemplar\_text = TRUE for a local,
unshared knit.

| topic | label | participant | segment | theta | tokens |
| --- | --- | --- | --- | --- | --- |
| t\_1 | 4 General Utility & Risk Appraisal | 299 | 299 | 1.00 | 60 |
| t\_1 | 4 General Utility & Risk Appraisal | 478 | 1296 | 0.99 | 57 |
| t\_1 | 4 General Utility & Risk Appraisal | 779 | 779 | 0.99 | 44 |
| t\_2 | 2 Conditional Acceptance & Clinical Authorship | 330 | 330 | 0.99 | 55 |
| t\_2 | 2 Conditional Acceptance & Clinical Authorship | 322 | 322 | 0.99 | 54 |
| t\_2 | 2 Conditional Acceptance & Clinical Authorship | 622 | 622 | 0.99 | 45 |
| t\_3 | 3 Relational Loss & the Limits of Simulated Contact | 348 | 348 | 1.00 | 86 |
| t\_3 | 3 Relational Loss & the Limits of Simulated Contact | 768 | 1586 | 0.99 | 56 |
| t\_3 | 3 Relational Loss & the Limits of Simulated Contact | 592 | 1410 | 0.99 | 46 |
| t\_4 | 1 Training Utility & Skill Rehearsal | 431 | 1249 | 0.99 | 28 |
| t\_4 | 1 Training Utility & Skill Rehearsal | 807 | 1625 | 0.99 | 27 |
| t\_4 | 1 Training Utility & Skill Rehearsal | 707 | 1525 | 0.99 | 26 |

# S5. Model selection note: fragmentation above *k* = 4

At higher *k*, manual semantic review showed core themes
fragmenting into overly granular sub-topics alongside poorly
interpretable residual topics. As an illustration, the patient model at
*k* = 8 splits engagement logistics into a narrow
reminder-focused topic.

Table S8. Example of fragmentation at k = 8 (patient corpus): a
narrow reminder/friction sub-topic (påminnelse = reminder, ihåg =
remember, irriterad = irritated) splits from the broader
engagement-logistics theme.

| rank | t\_8\_reminder\_topic |
| --- | --- |
| 1 | skriva |
| 2 | känna |
| 3 | irriterad |
| 4 | ihåg |
| 5 | påminnelse |
| 6 | jobbig |
| 7 | istället |
| 8 | kväll |

# S6. Exemplar and quotation verification (Table S11)

To guard against LDA grouping words by co-occurrence rather than
semantic relation, the top three most heavily loaded documents per
topic, subject to a floor of 15 content tokens because the
highest-loading very short segments were typically non-substantive, were
extracted and read in their original context before thematic labels were
assigned (S3.7 and S4.7).

In addition, every quotation presented in the manuscript was checked
against the fitted models. Table S11 reports, for each quotation, the
topic it was expected to load on given its placement in the manuscript,
the topic it actually loads on most strongly, and its full posterior
topic distribution. Quotations are presented in the manuscript in
English translation from the Swedish originals; the check below runs on
the Swedish source documents.

Table S11. Quotation verification. Expected topic is the topic
under which the quotation appears in the manuscript; observed topic is
the highest posterior loading of the source document. Theta is the full
document-topic distribution in model order (t\_1 to t\_4). Rank is the
position of the source document among all documents ordered by loading
on the expected topic; quotations were selected for readability rather
than by rank, so a high rank is not itself a discrepancy.

| Corpus | Quotation | Source | Expected | Observed | Match | Theta | Rank | Note |
| --- | --- | --- | --- | --- | --- | --- | --- | --- |
| Patient | direct follow-up (Theme 1) | p\_1030, segment 35 | t\_1 | t\_1 | yes | 0.99 / 0.00 / 0.00 / 0.00 | 1 |  |
| Patient | intensive at the beginning (Theme 1) | p\_1187, segment 394 | t\_1 | t\_1 | yes | 0.67 / 0.32 / 0.00 / 0.00 | 82 |  |
| Patient | unfathomable patience (Theme 2, embedded) | p\_1125, segment 212 | t\_3 | t\_3 | yes | 0.00 / 0.00 / 0.99 / 0.00 | 2 |  |
| Patient | explanatory models (Theme 2, embedded) | p\_1125, segment 213 | t\_3 | t\_3 | yes | 0.00 / 0.00 / 0.99 / 0.00 | 1 |  |
| Patient | school days (Theme 2) | p\_1026, segment 121 | t\_3 | t\_3 | yes | 0.00 / 0.00 / 0.99 / 0.00 | 4 |  |
| Patient | tools and everyday situations (Theme 2) | p\_1050, segment 414 | t\_3 | t\_3 | yes | 0.01 / 0.01 / 0.98 / 0.01 | 8 |  |
| Patient | not told friends (Theme 3) | p\_1014, segment 86 | t\_4 | t\_4 | yes | 0.00 / 0.00 / 0.00 / 0.99 | 3 |  |
| Patient | cast in a mold (Theme 3) | p\_1065, segment 159 | t\_4 | t\_4 | yes | 0.00 / 0.00 / 0.00 / 0.99 | 4 |  |
| Patient | a natural end to it (Theme 4) | p\_1030, segment 27 | t\_2 | t\_2 | yes | 0.00 / 0.99 / 0.00 / 0.00 | 2 |  |
| Patient | evening / adapted myself (Theme 4) | p\_1030, segment 22 | t\_2 | t\_2 | yes | 0.11 / 0.88 / 0.01 / 0.01 | 55 |  |
| Psychologist | ID 178, scenario 2 |  | t\_4 | t\_4 | yes | 0.14 / 0.00 / 0.00 / 0.85 | 282 |  |
| Psychologist | ID 431, scenario 2 |  | t\_4 | t\_4 | yes | 0.00 / 0.00 / 0.00 / 0.99 | 1 |  |
| Psychologist | ID 807, scenario 2 |  | t\_4 | t\_4 | yes | 0.00 / 0.00 / 0.00 / 0.99 | 2 |  |
| Psychologist | ID 322, scenario 1 |  | t\_2 | t\_2 | yes | 0.00 / 0.99 / 0.00 / 0.00 | 2 |  |
| Psychologist | ID 330, scenario 1 |  | t\_2 | t\_2 | yes | 0.00 / 0.99 / 0.00 / 0.00 | 1 |  |
| Psychologist | ID 203, scenario 1 |  | t\_2 | t\_2 | yes | 0.00 / 0.89 / 0.06 / 0.06 | 188 |  |
| Psychologist | ID 592, scenario 2 |  | t\_3 | t\_3 | yes | 0.00 / 0.00 / 0.99 / 0.00 | 3 |  |
| Psychologist | ID 768, scenario 2 |  | t\_3 | t\_3 | yes | 0.00 / 0.00 / 0.99 / 0.00 | 2 |  |
| Psychologist | ID 348, scenario 1 |  | t\_3 | t\_3 | yes | 0.00 / 0.00 / 1.00 / 0.00 | 1 |  |
| Psychologist | ID 299, scenario 1 |  | t\_1 | t\_1 | yes | 1.00 / 0.00 / 0.00 / 0.00 | 1 |  |
| Psychologist | ID 478, scenario 2 |  | t\_1 | t\_1 | yes | 0.99 / 0.00 / 0.00 / 0.00 | 2 |  |
| Psychologist | ID 769, scenario 2 |  | t\_1 | t\_4 | no | 0.13 / 0.03 / 0.13 / 0.71 | 474 | presented in the Theme 4 section but attributed in the text to the training topic |

21 of 22 quotations load most strongly on the topic under which they
are presented. The single exception is deliberate and is noted in the
table.

# S7. Post-submission audit (Table S12)

After the manuscript was submitted, both topic models were refitted
from the raw data files in a clean session and every quotation in the
manuscript was rechecked against the refitted models. The audit had two
parts.

First, reproducibility. Table S12 compares the refitted topic
prevalences and top-ten term lists against the values reported in the
manuscript and in Tables S2 and S6 above. The refit reproduced both
solutions exactly, so the analysis is reproducible from the raw data
with the code in this document.

Second, quotation placement. The item-level check is Table S11. One
patient quotation printed under Theme 2, concerning tools and previously
avoided situations, was found to load on Theme 1 (Analytic Engagement
and Situational Insight) rather than on Theme 2, and ranked far down the
ordering of documents loading on Theme 2. It was replaced with the
segment now reported in Table S11, which makes the same point and loads
on Theme 2 as intended. One psychologist quotation (ID 769, scenario 2)
is presented in the Theme 4 section while loading on the training topic;
this is deliberate and the manuscript text attributes it to that topic
explicitly, so it is recorded as a note rather than as a correction. The
issue was therefore confined to the placement of a single illustrative
quotation and affects neither the topic models nor any reported
result.

Table S12. Reproduction check. Published values are those
reported in Tables S2 and S6 and in the manuscript; refitted values were
computed by fitting both models again from the raw data in this knit.
Prevalence is in per cent; the top-terms column compares the full
ten-term lists.

| Model | Topic | Published | Refitted | Prevalence | Top terms |
| --- | --- | --- | --- | --- | --- |
| Patient | t\_1 | 25.1 | 25.1 | match | match |
| Patient | t\_2 | 27.6 | 27.6 | match | match |
| Patient | t\_3 | 22.3 | 22.3 | match | match |
| Patient | t\_4 | 25.0 | 25.0 | match | match |
| Psychologist | t\_1 | 21.2 | 21.2 | match | match |
| Psychologist | t\_2 | 24.6 | 24.6 | match | match |
| Psychologist | t\_3 | 22.9 | 22.9 | match | match |
| Psychologist | t\_4 | 31.2 | 31.2 | match | match |

Prevalence mismatches: 0 | top-term list mismatches: 0

# Supplementary Methods

## M1. Patient interviews

Participants randomized to the AI-CBT or AI-PDT arm were shown a
written electronic invitation to an interview study when completing the
post-treatment assessment. The waitlist group was not shown this
invitation. Twenty participants expressed interest and were contacted
through the Iterapi study platform. Because response was low, text
messages and email followed after one week. Fourteen replied and
remained interested; three preferred a text-only interview and were not
included because interviews were conducted by telephone. Eleven patients
were interviewed without compensation. During recruitment, the team
aimed to obtain 15 interviews. The final sample was limited by the
availability of participants willing to be interviewed; no formal
saturation assessment was used as a stopping rule.

Catharina Lutovsky, a female psychology student in a five-year
clinical psychology programme with a psychodynamic orientation,
conducted the interviews in May and June 2025. She had received
instruction in qualitative research interviewing and had some previous
experience of conducting such interviews. She had not treated the
participants and had no prior personal or professional relationship with
them. Participants were told that she was involved in the research and
wished to explore their experiences of AI treatment; the interview was
presented as research rather than treatment.

She conducted the telephone interviews from her home to provide a
quiet environment. No other person was present with the interviewer; the
presence of others at the participant end was not systematically
recorded. Each participant was interviewed once. Interviews were
recorded using a dictaphone and lasted a mean of 20 minutes (range 12 to
28). Written consent was obtained before the interview and oral consent
at its start. Some notes were taken during and after interviews, but
field notes were not collected systematically. Further notes were made
during transcript reading. Transcripts were not returned to participants
for correction, and participants were not asked to review the
findings.

The interview guide was developed with assistance from Per Carlbring.
Gemini 2.5 Pro was used as an adversarial reviewer (“devil’s advocate”)
to critically review the interview guide. It contained 11 numbered open
questions, follow-up prompts, and an open closing question. A pilot
interview with a student within the STePS-AI project was conducted at
the university to test the questions and practice interviewing; no
question changes were considered necessary. The original Swedish guide
and an English translation for author review are provided in
Supplementary interview guide, Sections A and B.

Recordings were transcribed in autumn 2025 using Whisper.
Transcription errors and omissions associated with poor recording
quality required manual correction. The interviewer had used AI as a
study aid but had no prior experience of AI-delivered therapy. She
initially viewed it as a possible complement or entry point to therapy
rather than a replacement for a human therapist and described how her
initial skepticism influenced her reading of the interviews. Earlier
manual coding did not inform the present topic interpretation.

## M2. Psychologist survey

The parent questionnaire was developed by Kristofer Vernmark and
revised following feedback from Gerhard Andersson and Per Carlbring. It
comprised nine sections and 31 items, with five additional conditional
items, for a maximum of 36. The present analysis concerns the two
open-ended AI-scenario prompts; the parent report addresses quantitative
questionnaire data. All questions were administered in Swedish through
Iterapi, and the questionnaire took approximately 15 minutes to complete
(Vernmark et al., 2026). The draft was revised after expert feedback; no
separate participant pretest was performed.

The online questionnaire was distributed in January-February 2026 and
remained open for 41 days. Using convenience sampling, we recruited
members of the Swedish psychology association (psychology master’s
students, internship psychologists, and licensed psychologists) via the
association’s emails (n = 12,603) and social media channels (n = 5,900),
with expected overlap. To prevent non-psychologist responses, social
media links routed to the association website, where access required
Swedish BankID verification. Recruitment included three emails (plus two
reminders) and multiple social media posts and ads. All materials
described the study purpose, anonymity, and expected completion time,
and informed consent was required. No incentives were offered (see
Vernmark et al., 2026).

The scenario module was offered as an optional continuation of the
parent questionnaire. Questions and the two scenarios were presented in
a fixed order without randomization or counterbalancing. The scenario
free-text fields were optional and could be left blank. There was no
separate sample-size calculation for this secondary analysis: sample
size was determined by available responses meeting the selection and
preprocessing criteria.

No dedicated analysis of nonresponse against the invited population
and no population weighting were performed for the free-text analysis. A
reliable denominator of unique invitees and a separately verified count
completing the entire questionnaire were unavailable, so no conventional
overall survey response rate is reported for this analysis. The parent
report’s 947 complete cases concern its quantitative analysis items, not
necessarily completion of the entire questionnaire.

Table 2 describes the 818 respondents retained before document
preprocessing; 808 contributed at least one analyzable document. Valid
age was unavailable for 24 of the 818 respondents and valid year of
licensure for 118; these values were treated as missing.

## M3. Text analysis and interpretation

Preprocessing of the native Swedish text was conducted in R (R Core
Team, 2021) using the textmineR (Jones et al., 2025) and udpipe
(Wijffels & Straka, 2026) packages. To address the complexities of
Swedish compound words and verb conjugations without losing semantic
meaning, standard stemming was avoided. Instead, text was lemmatized
using a Swedish dependency parser to convert words to their base
grammatical roots (e.g., transforming ‘kändes’ to ‘känna’). We applied
lowercasing, removed punctuation and numbers, and applied a single
lemma-aware stop list covering standard stopwords, conversational and
evaluative fillers, and abbreviation artifacts (e.g., ‘liksom’, ‘bra’,
‘t.ex.’), identically across both corpora. Text was translated from
Swedish to English only after analysis was complete, so that the models
operated on the original language and no semantic nuance was lost to
translation beforehand.

A Document-Term Matrix (DTM) was created capturing unigrams, bigrams,
and trigrams. Infrequent terms (appearing in fewer than two documents)
were removed to reduce noise. The LDA model was fitted using Gibbs
sampling (2000 iterations; hyperparameters α = 0.1, β = 0.5). To
determine the optimal number of topics (k), models ranging from k = 2 to
k = 15 were evaluated using average probabilistic coherence (Tables S3
and S5). The trajectory did not sharply favor any single k for the
patient corpus (maximum at k = 7, with k = 4 within 0.0013), and for the
psychologist corpus the maximum fell at k = 10, above the solution we
retained. We did not select k by maximizing coherence, because
probabilistic coherence rewards granular, near-duplicate topics in short
text (Chang et al., 2009). Instead, k was selected on interpretability
and lexical distinctness. Manual semantic review of the higher-k
solutions showed core clinical themes fragmenting into overly granular
sub-topics alongside poorly interpretable residual ones; at k = 8, for
example, engagement logistics in the patient corpus split off a narrow
reminder-focused topic (Table S8). At k = 4 the psychologist topics
remained lexically distinct (Table S7). A model of k = 4 was selected.
Finally, to address the limitation of LDA grouping words by
co-occurrence rather than strict semantic relation, the mathematical
output was manually validated. The top three most heavily loaded
documents (transcripts) for each topic were extracted, subject to a
floor of 15 content tokens, because the highest-loading very short
segments were typically non-substantive, and the highest-probability
terms were reviewed in their original qualitative context to define the
final thematic labels.

We use theme to denote the interpretive label assigned to an LDA
topic. Per Carlbring and Jón Ingi Hlynsson reviewed the model output and
high-loading excerpts in context and agreed on interpretations through
discussion. There was no independent double coding or inter-rater
reliability statistic. Earlier manual coding did not inform these
interpretations. No separate formal negative-case analysis was
performed.

All models were estimated with a fixed random seed; coherence refers
to textmineR’s probabilistic coherence. N-grams were included as
additional tokens alongside their constituent unigrams, so multiword
expressions (e.g., social\_ångest) could surface as terms in their own
right. Topic prevalence was operationalized throughout as the mean
posterior document-topic probability, weighting documents equally, and
is also termed mean topic weight below. Prevalence values are therefore
model-based topic weights, not proportions of participants or documents
assigned to a single topic. The patient estimates use 390 analyzable
segments from 11 participants. The psychologist estimates use 1,551
analyzable documents from 808 respondents, comprising 783 Digital Twin
and 768 Virtual Patients documents. The psychologist solution re-emerged
under alternative random seeds (mean top-term overlap of 75-88%,
comparing lists of ten terms across four matched topics). The patient
solution, fitted on a far smaller corpus, was only moderately
seed-stable (mean overlap 52-60%, using the same ten-term lists and
four-topic matching), which underscores the role of the exemplar-based
validation described above; as a further check on this corpus, a biterm
topic model, designed for short texts (Yan et al., 2013), was fitted as
a benchmark. The submitted analysis report states that quotations were
checked against topic assignments. The item-level verification is
already reported in the analysis supplement, which is submitted with the
manuscript. The analysis code, document-flow accounting for both
corpora, coherence-by-k values, lexical-distinctness matrices and
seed-stability results are provided in the existing analysis
supplement.

# Supplementary patient quotations

These additional examples have been moved intact from the submitted
Results to keep the main text within the journal’s word limit. The main
text retains an example for each patient theme.

## Patient Theme 1: Analytic Engagement and Situational Insight

*“It was kind of nice that it could be fairly intensive,
especially at the beginning of the process. I imagine that when you go
to therapy otherwise, there are longer breaks, you know. That was an
advantage, in a way; you could take several days in a row and really get
into it, and feel that it was a process that was constantly moving
forward at my own pace, somehow.”*

## Patient Theme 2: Deep Clinical Processing and Self-Understanding

*“I’ve actually sought support a bit when I’ve been overcome by
intense fatigue… when I didn’t know what to do. Like,”What should I do
here? How do I move forward?” That’s when I’ve turned to [the AI
chatbot]. I’ve asked for advice and for suggestions on how to approach
different situations, and asked [the AI chatbot] to provide things like
social scripts. [It] simply given me the tools I need.”*

## Patient Theme 3: Relational Authenticity, Stigma and Human-AI Comparison

*“The AI is very… well, it’s like it’s cast in a mold, I think.
It’s maybe a bit too… well, what should I say, upbeat in a way. It feels
a bit artificial, you notice that it is an AI. It uses the same kind of
language, there aren’t many nuances. With a human, you get a completely
different interaction. You have body language, you have the human
relationship that is created between… well, when you have a conversation
with another person. I think you can tell with the AI, that it is an
AI.”*

## Patient Theme 4: On-Demand Access and Engagement Logistics

*“I feel that I have put my time, my energy into this for the day,
then I don’t want to do it in the evening too, so when I try, I’ve
adapted myself a bit accordingly… I could just as easily have said that
doing it in the evening doesn’t suit me, I could have done that. But it
works in the evening too, so I chose to usually schedule it then
instead.”*

# Supplementary interview guide

The Swedish interview guide is reproduced in Section A. It contains
11 numbered questions, follow-up prompts and an open closing question.
Section B contains the English translation. Interviews were conducted in
Swedish.

## A. Original Swedish guide

1. Berätta om hur du först kom i kontakt med AI-terapin och vad som
   gjorde att du ville prova den.
2. Hur skulle du beskriva dina förväntningar innan du började?
3. Och fanns det något du oroade dig för innan start?
4. Kan du ta mig igenom ett typiskt tillfälle när du använde
   AI-chatten?

- Följdfrågor: Vad gjorde du just då? Vad hände i samtalet? Hur kändes
  det efteråt?

5. På vilket sätt upplevde du att AI:n försökte förstå dig och dina
   problem?

- Följdfrågor: I vilken utsträckning kände du dig faktiskt förstådd av
  AI:n? Kan du ge exempel?

6. Finns det särskilda stunder då terapin kändes extra
   hjälpsam? Berätta om ett sådant tillfälle.
7. Och ett tillfälle då den inte fungerade lika bra?
8. Hur upplevde du AI-terapins tillgänglighet dygnet runt?

- Följdfrågor: När använde du den oftast? Hur länge brukade du stanna
  i chatten? Vad fick dig att avsluta chatten?

9. Kände du att du kunde vara helt öppen med AI-terapeuten? Kan du ge
   exempel?
10. Om du jämför med att prata med en mänsklig terapeut – vad ser du för
    likheter och skillnader?
11. På vilket sätt, om alls, har AI-terapin påverkat din sociala ångest
    i vardagen?

Avslutning: Finns det något mer du vill lägga till som vi inte pratat
om men som känns viktigt?

## B. English translation

1. Tell me how you first came into contact with AI therapy and what
   made you want to try it.
2. How would you describe your expectations before you started?
3. And was there anything you were worried about before starting?
4. Can you take me through a typical occasion when you used the AI
   chat?

- Follow-up questions: What were you doing at the time? What happened
  in the conversation? How did you feel afterwards?

5. In what ways did you feel that the AI tried to understand you and
   your problems?

- Follow-up questions: To what extent did you actually feel understood
  by the AI? Can you give examples?

6. Were there particular moments when the therapy felt especially
   helpful? Tell me about one such occasion.
7. And an occasion when it did not work as well?
8. How did you experience the round-the-clock availability of AI
   therapy?

- Follow-up questions: When did you use it most often? How long did
  you usually stay in the chat? What made you end the chat?

9. Did you feel you could be completely open with the AI therapist? Can
   you give examples?
10. If you compare it with talking to a human therapist, what
    similarities and differences do you see?
11. In what ways, if any, has AI therapy affected your social anxiety in
    everyday life?

Closing question: Is there anything else you would like to add that
we have not talked about but that feels important?

# References

Chang, J., Gerrish, S., Wang, C., Boyd-Graber, J., & Blei, D.
(2009). Reading tea leaves: How humans interpret topic models.
*Advances in Neural Information Processing Systems*, 22.

Jones, T., Doane, W., & Attbom, M. (2025). *textmineR:
Functions for text mining and topic modeling* (Version 3.0.6)
[Computer software].

R Core Team. (2021). *R: A language and environment for
statistical computing*.

Vernmark, K., Carlbring, P., & Andersson, G. (2026). *Adoption
of Generative AI in the Psychology Profession: Findings from a National
Survey of Over 1,000 Swedish Psychologists (Preprint)*. JMIR Mental
Health. https://doi.org/10.2196/preprints.107511

Wijffels, J., & Straka, M. (2026). *udpipe: Tokenization,
parts of speech tagging, lemmatization and dependency parsing with the
“UDPipe” “NLP” toolkit* (Version 0.8.16) [Computer software].

Yan, X., Guo, J., Lan, Y., & Cheng, X. (2013). A biterm topic
model for short texts. *Proceedings of the 22nd International
Conference on World Wide Web*, 1445–1456. https://doi.org/10.1145/2488388.2488514
